# The Value and the Need: Preparing BSN Students for Long-Term Care Employment

**DOI:** 10.1093/geroni/igaf122.3673

**Published:** 2025-12-31

**Authors:** Kimberly Delgado, Donna Roberson, Jean Matthews

**Affiliations:** East Carolina University College of Nursing, Greenville, North Carolina, United States; East Carolina University, Greenville, North Carolina, United States; East Carolina University, Ahoskie, North Carolina, United States

## Abstract

Long-term care (LTC) settings consistently struggle to meet staffing demands, especially adequate registered nurse (RN) coverage. With an increasing US older adult population, the need for LTC is expected to grow. The Center for Medicare and Medicaid made staffing recommendations to increase RNs employed in LTC. BSN nursing education focuses on general nursing knowledge heavily centered on acute care settings, not specific training in LTC. With support from the Carolina Geriatric Workforce Enhancement Program grant, the team developed an innovative course with tuition support for five students selected from a competitive pool of thirteen applicants. The course provided skills and knowledge focused on LTC settings, care of older adults, and 24 hours of clinical experience in LTC. Qualitative inquiry throughout the course included pre/post course discussion and reflection on the experience. Analysis indicated that the students developed an improved attitude to LTC work, including increased appreciation for the RN role in LTC. The students found the course to be comprehensive, yet reasonable to accomplish with other educational demands of their BSN program. All five students indicated intention to seek LTC employment, if not upon graduation, at some point in their career. One student said that she could “really see the value and need” for BSN RNs in residential care. To meet the demand for BSN RNs in LTC, such programming is essential in BSN programs (eventually nationwide). The course will be repeated with ongoing data collection and tracking of employment sites upon participant graduation.

